# Disseminated Coccidioidomycosis

**DOI:** 10.3201/eid1101.040613

**Published:** 2005-01

**Authors:** Cheng-Yi Wang, Jih-Shuin Jerng, Jen-Chung Ko, Ming-Feng Lin, Cheng-Hsiang Hsiao, Li-Na Lee, Po-Ren Hsueh, Sow-Hsong Kuo

**Affiliations:** * National Taiwan University Hospital, Taipei, Taiwan; † Hsin-Chu General Hospital, Hsinchu, Taiwan

**Keywords:** letter, coccidioidomycosis, disseminated, Taiwan

**To The Editor**: Coccidioidomycosis, an infection caused by the dimorphic fungus *Coccidioides immitis*, is endemic in the southwestern United States, parts of Mexico, and Central and South America (*1*). Patients with *C. immitis* infection may have chronic pneumonia, fungemia, and extrapulmonary dissemination to skin, bones, meninges, and other body sites. The clinical features of coccidioidomycosis may mimic those of melioidosis, penicilliosis marneffei, and tuberculosis, which are commonly seen in some southeastern Asian countries, including Taiwan.

A previously healthy, 71-year-old retired gynecologist from Taiwan, visited Los Angeles in August 2003 and traveled to the San Joaquin Valley in November 2003. He had smoked 1 package of cigarettes daily for 50 years. He noted fever 5 days before returning to Taiwan on December 1, 2003. He came to a local hospital on December 4 with a temperature of 39°C and a history of 1 month of night sweats, productive cough, and weight loss of 10 kg. Chest radiograph showed diffuse nodular lung lesions bilaterally (Figure, panel A). His leukocyte count was 16.65 x 10^9^/L (neutrophils 85.6%, lymphocytes 6.2%), and C-reactive protein was 21.5 mg/dL (reference value, <0.8 mg/dL). Empiric antimicrobial drugs (amoxicillin/clavulanic acid and ciprofloxacin) and antituberculosis therapy (isoniazid, rifampin, ethambutol, and pyrazinamide) were administered. Blood and sputum specimens were negative for bacteria; HIV antibody test results were negative, but the fever persisted. A follow-up chest film showed a left pleural effusion. The pleural effusion aspirate was exudative with 3.6 x 10^9^/L leukocytes (73% neutrophils). Computed tomographic scan of the patient’s chest showed collapse of the left lower lung with central necrosis, bilateral pleural effusions, and mediastinal lymphadenopathy. Pleural biopsy by video-assisted thoracoscopic surgery showed no evidence of malignancy, but heavy lymphoplasmacytic infiltration and chronic necrotizing granulomatous inflammation were found (Figure, panel C). On December 17, 2003, 30 mg/day prednisolone orally was prescribed for intermittent fever. Biopsy material and cultures of blood samples taken at admission grew an unidentified mold, which was also isolated from the biopsy wound. The patient was discharged afebrile from the hospital on January 20, 2004. The fever recurred, with a disturbance in consciousness on January 25, 2004. Computed tomographic scan of the brain revealed no obvious organic lesions. He was referred to our hospital on January 26, 2004.

**Figure:**
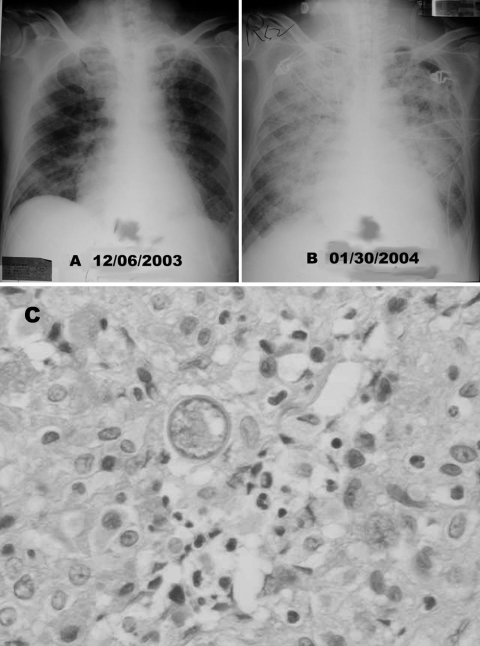
A) Chest radiograph shows diffuse nodular lesions in both lungs. B) Chest radiographic scan taken 2 months later shows coalescence of nodular shadows and almost complete white-out of bilateral lung fields. C) Hematoxylin and eosin staining of the wound specimen from pleural biopsy site showed spherules of *Coccidioides immitis* and chronic necrotizing granulomatous inflammation (400x).

After the patient was admitted, fever persisted and respiratory distress worsened rapidly. He developed severe headache, seizures, and loss of consciousness. He was transferred to the intensive care unit for aggressive management of acute respiratory distress syndrome and deterioration of renal function. Chest radiograph showed coalescence of nodular shadows and almost complete white-out of bilateral lung fields (Figure, panel B). Meropenem, antituberculosis agents, and intravenous voriconazole, 200 mg every 12 hours, were administered.

Both the unidentified mold, which was sent to our hospital for further identification, and a mold cultured from the previous biopsy wound at our hospital were identified as *C. immitis* by their characteristic gross and microscopic morphotypes in standard slide cultures incubated at 28°C for 10 days. Hematoxylin and eosin staining of the biopsied tissue showed many spherules.

Lumbar puncture was performed on January 30, 2004, and showed an elevated opening pressure of 380 cm H_2_O and a few destructed large spherules in the cerebrospinal fluid (CSF). However, cultures of CSF were negative for bacteria and fungi. After the diagnosis of disseminated coccidioidomycosis (pneumonia, fungemia, and meningitis), voriconazole was replaced by intravenous fluconazole, 400 mg/day. The patient’s intensive care course was complicated by *Pseudomonas* pneumonia and repeated episodes of upper gastrointestinal bleeding. A second lumbar puncture was conducted on February 13, 2004, and also showed an elevated opening pressure (290 cm H_2_O). Uncontrolled coccidioidomycosis meningitis was suspected, and intrathecal amphotericin B treatment was planned. Refractory shock with bradycardia developed when an intrathecal catheter was implanted. The patient did not respond to therapy and died on February 16, 2004. The MIC of fluconzole for the *C. immitis* isolate was 48 μg/mL, and the MIC of amphotericin B was found to be 1 μg/mL for by using the Etest (ABiodisk, Solna, Sweden) according to manufacturer’s information.

This case is the first to be reported of disseminated coccidioidomycosis with fulminant pneumonia, fungemia, and meningitis reported from Taiwan (*2*). Review of the patient’s travel history and clinical course indicated that the *C. immitis* was acquired in California and that the initial manifestations had begun before the patient returned to Taiwan. Coccidioidomycosis is commonly diagnosed in disease-endemic areas but frequently overlooked in disease-nonendemic areas because of a low index of suspicion among physicians. The interval from onset of symptoms to disease diagnosis was relatively long (*3*). Our patient had chills, productive cough, weight loss, and night sweats followed by fever as the initial manifestations of this infection. These symptoms had been most frequently reported in previous coccidioidomycosis cases (*4*). Radiographic scans of the patient initially showed diffuse reticular lesions, followed by pleural effusion and consolidation. This clinical course was also fully compatible with those of previously reported cases (*4*). However, the clinical manifestations of chronic pneumonia with pleural effusion, the initial partial response to steroid treatment, and the delay in recognizing the mold contributed to delayed diagnosis of this disease.

The isolate was not susceptible to fluconazole (MIC 48 μg/mL). Although the National Committee for Clinical Laboratory Standards does not have a standard susceptibility method and MIC breakpoint of fluconazole for defining susceptibility against *C. immitis*. Fluconazole has been recommended as a drug of choice for treating meningal coccidioidomycosis, particularly in patients with underlying renal disease or with disease-associated renal function deterioration (*4*,*5*). Immunocompromise secondary to steroid use, as well as resistance of the isolate to fluconazole, may have contributed to treatment failure in this patient.

With increasing international travel, physicians should consider those diseases that are endemic in regions where their patients have traveled. In addition to tuberculosis, melioidosis, and penicilliosis marneffii, coccidioidomycosis should be included in the differential diagnosis of chronic pneumonia in Taiwan, considering the number of residents who travel. Only then can prompt microbial investigations be conducted to accurately diagnosis and determine the appropriate antifungal treatment.
